# Evaluation of Mechanochemically Prepared CePO_4_∙H_2_O Nanoparticles as UV Filter for Photoprotective Formulations

**DOI:** 10.3390/molecules30020405

**Published:** 2025-01-18

**Authors:** Stanislav Kurajica, Filip Brleković, Sabina Keser, Goran Dražić, Katarina Mužina, Vanesa Mihajlović

**Affiliations:** 1 Faculty of Chemical Engineering and Technology, University of Zagreb, Marulićev trg 19, HR 10000 Zagreb, Croatia; fbrlekovi@fkit.unizg.hr (F.B.); kmuzina@fkit.unizg.hr (K.M.); vanesa.mihajlovic@gmail.com (V.M.); 2 Faculty of Pharmacy and Biochemistry, University of Zagreb, A. Kovačića 1, HR 10000 Zagreb, Croatia; sabina.keser@gmail.com; 3 National Institute of Chemistry, Hajdrihova 19, SI-1001 Ljubljana, Slovenia; goran.drazic@gmail.com

**Keywords:** cerium phosphate, mechanochemical synthesis, UV filter, cytotoxicity, photoprotection

## Abstract

Rhabdophane, CePO_4_∙H_2_O, nanoparticles were prepared by mechanochemical synthesis with different durations and thoroughly characterized by various characterization techniques. X-ray diffraction analysis showed that the optimal synthesis duration was 15 min, since, in this case, pure rhabdophane is obtained, without traces of contamination by the vessel material. The size of the obtained nanoparticles, as determined from high-resolution transmission electron microscopy images, was around 5 nm. According to UV-Vis diffuse reflectance spectroscopy results, rhabdophane nanoparticles show transparency to visible light and high absorption in the UV region, with a direct bandgap of 3.1 eV. The photocatalytic activity in the Castor oil degradation process and the cytotoxicity for human skin cells were determined and compared to commercial TiO_2_ nanoparticles, with rhabdophane nanoparticles exhibiting superior properties. Small particle size, purity, absorption in the UV range, transparency to visible light, low photocatalytic activity, and low cytotoxicity indicated the possibility of prepared rhabdophane application as an inorganic UV filter in photoprotective formulations.

## 1. Introduction

Excessive exposure to the sun can have unwanted and dangerous consequences, such as sunburn, accelerated skin aging, immunosuppression, and skin tumors [1]. The protection of skin from the harmful effects of the sun, particularly UV radiation, is usually achieved by applying a suitable sunscreen. The most important compounds in sunscreen formulations are UV filters [2], which can be organic and inorganic [3]. The action mechanism of organic UV filters is exclusively the absorption of UV radiation. Their downside is the possibility of absorption through the skin, which leads to a reduction in the effectiveness of protection and the possibility of skin irritation [2]. On the other hand, inorganic filters work by reflecting, scattering, or absorbing UV radiation. Their downsides are catalytic activity, which can lead to chemical changes in other components of the cream, and a visible white film on the skin, which is the result of reflection and scattering [4]. The most commonly used inorganic UV filters are zinc oxide, ZnO, and titanium oxide, TiO_2_, these being the only registered and approved filters in the EU and USA [5]. Both compounds have considerable photocatalytic activity and catalyze the formation of many reactive oxygen species, which can lead to sunscreen instability and, more importantly, have a cytotoxic effect, cause skin damage, and the appearance of tumors. Finally, both materials possess a high refractive index, leading to pronounced phenomena of reflection and scattering in the visible light spectrum [6]. Therefore, efforts are being made to find a suitable substitute for these compounds with reduced or negligible photocatalytic activity, a lower refractive index, and smaller particle size.

Particular attention is paid to cerium-based materials due to their wide distribution and favorable properties. Cerium phosphate, CePO_4_, is a potentially suitable material for this purpose due to its high absorption of UV radiation, lower refractive index, and reduced photocatalytic activity compared to titanium dioxide and zinc oxide [2,4]. Cerium phosphate occurs in two forms: hexagonal hydrated form rhabdophane, CePO_4_⋅nH_2_O, and monoclinic anhydrous monazite, CePO_4_ [7]. Besides being used as a UV filter, cerium phosphate has also been investigated for its optical (fluorescence), chemical (ion exchange), photocatalytic, refractive, and anticorrosive properties [8,9,10,11].

A significant trend in the preparation of sun protection products is avoiding the formation of a white film on the skin, primarily for aesthetic reasons, which is achieved by using nanoparticles. When the particles are in the nano-size range or at least significantly smaller than the wavelengths of visible light, reflection and scattering on these particles are insignificant [5]. Therefore, the application of creams containing sufficiently fine particles of an inorganic UV filter does not impair transparency in the visible area (the white film does not form) [3]. At the same time, due to the absorption that occurs during the transition of electrons from the valence band to the conduction band of the inorganic UV filter, the cream provides good protection against UV radiation.

There are various techniques for the preparation of cerium orthophosphates, such as hydrothermal [12], coprecipitation [8,9], microemulsion [3], sol-gel [11,13], mechanochemical [10], sonochemical, [14,15], microwave-assisted method [16], etc. The modern approach to chemical synthesis is to make it environmentally friendly by reducing the energy consumption, minimizing the use of organic solvents, limiting the generation of toxic waste, etc. Synthesis methods that meet these criteria are classified as green methods; one such method is mechanochemical synthesis. It is a synthesis approach in which chemical transformations are induced by the application of mechanical energy, usually by means of high-energy grinding in a ball mill. The method does not require the use of solvents but, rather, optimizes reactant utilization and enhances product selectivity, often minimizing or even avoiding by-products. Additionally, it typically reduces reaction times and energy consumption. This approach can also produce products with sizes in the nano range [17].

The goal of this investigation was to develop a mechanochemical synthesis method for nanocrystalline cerium phosphate. Cerium chloride heptahydrate and sodium phosphate dodecahydrate were used as precursors, with sodium chloride serving as an inert diluent, enabling the separation of produced nanoparticles and preventing their subsequent growth. Another goal was the characterization of the obtained material to test if it meets the requirements of effective sun protection: ability to reflect UV light, no photocatalytic activity, and no cytotoxic effects.

## 2. Results and Discussion

**Figure 1:**
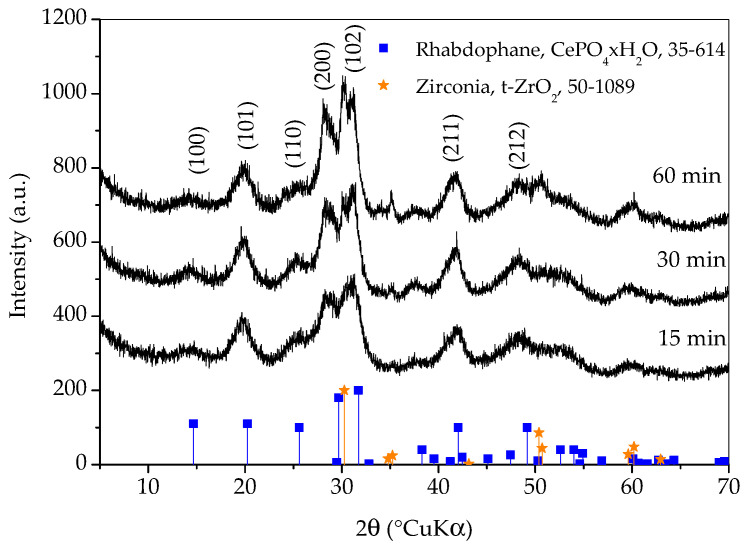
X-ray diffraction patterns of samples prepared with different durations of synthesis. Only the main rhabdophane peaks are labeled.

Figure 1 shows X-ray diffraction patterns of samples prepared with different synthesis durations. All three diffraction patterns show very broad peaks, which indicates the nanocrystalline character of the obtained phase(s). The diffraction patterns of all three samples closely match the diffraction pattern of rhabdophane, CePO_4_∙H_2_O, ICDD PDF No. 35-614. Hydrated orthophosphate, rhabdophane, crystallizes in the hexagonal symmetry, although the recent literature opts for monoclinic structure [18]. Even though the peaks are very broad, careful observation of the diffraction patterns reveals that the positions of the diffraction maxima do not agree perfectly with the literature data. This is best perceived for the (200) peak which should appear at 29.68 °(2θ) and it is notably shifted to lower angles. Looking for an explanation, one would first think of the presence of an additional phase such as monoclinic monazite, CePO_4_, ICDD PDF No. 32-199. On the other hand, such a shift could be the consequence of the increase in lattice parameters [3]. Lattice expansion in nanoparticles has been observed for many ionic compounds and, according to Diehm et al. [19], the key reason for lattice expansion is negative surface stress. Unfortunately, the broadness of the peaks and their overlapping made an exact calculation of the lattice parameters impossible, so this inconsistency will be resolved using other techniques. A rhabdophane crystallite size of 3.9 ± 0.5 nm was calculated on the basis of (101) reflex (at 20.25 °2θ), which was chosen as the most appropriate due to peaks’ overlapping.

Diffraction lines of tetragonal zirconia, t-ZrO_2_, ICDD PDF No. 50-0189, are clearly visible on the pattern of the sample whose synthesis lasted for 60 min. These lines are weaker on the pattern of the sample whose synthesis took 30 min and imperceptible on the pattern of the sample whose synthesis took 15 min. It is obvious that a shorter synthesis time avoids excessive wear of the vessel and balls, as well as the contamination of the sample with ZrO_2_. On the other hand, there are no peaks characteristic for reactants in any of the patterns and the intensities of the rhabdophane peaks do not differ significantly, so it can be assumed that the reaction is complete. Therefore, only the sample whose synthesis lasted 15 min was further investigated, since the contamination and wear of the vessel and balls were negligible.

**Figure 2:**
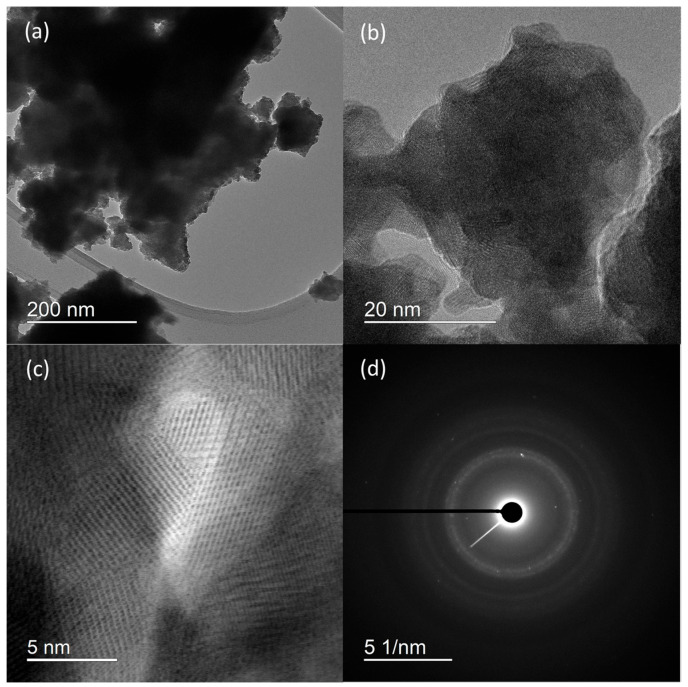
HRTEM micrographs at different magnifications (**a**–**c**) and SAED pattern (**d**) of sample prepared by 15 min synthesis.

**Figure 3:**
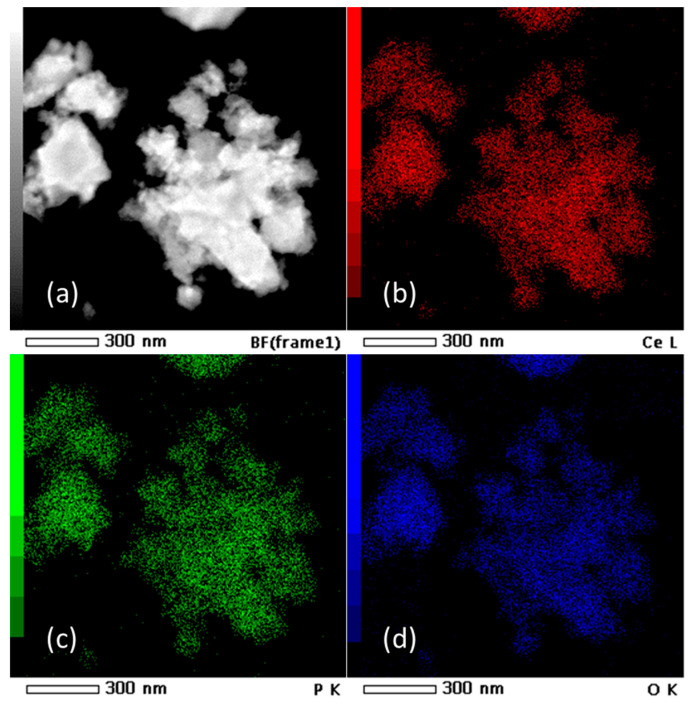
TEM micrograph (**a**) and EDS mapping of Ce (**b**), P (**c**), and O (**d**) in the sample prepared by 15 min synthesis.

HRTEM micrographs (Figure 2) suggest that the prepared material consist of particle agglomerates of spherical nanosized particles (Figure 2a) with estimated size around 5 nm (Figure 2b), which is very close to the value calculated for the crystallite size. In the HRTEM image (Figure 2c), lattice fringes can be clearly observed. The most commonly measured distance between adjacent fringes was 3.0 nm, corresponding to the interplanar distances of the (200) planes of rhabdophane. From the diameters of the diffraction rings in the corresponding SAED pattern (Figure 2d), the interplane spacings were calculated to be 0.30, 0.28, 0.21, and 0.19 nm, which can be indexed as the (200), (102), (211), and (212) planes of rhabdophane. Therefore, the results of the HRTEM analysis indicate the presence of solely rhabdophane. EDS mapping (Figure 3) reveals completely homogeneous distribution of cerium, phosphorus, and oxygen in the prepared sample.

**Figure 4:**
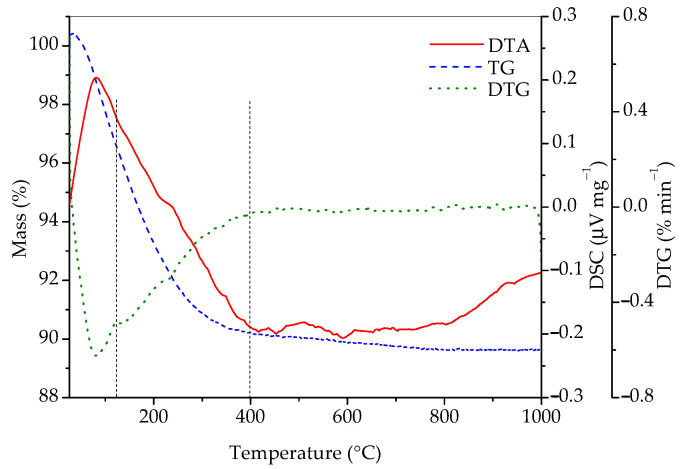
DTA, TG, and DTG curves of sample prepared by 15 min synthesis.

In order to gain additional information on its composition, the sample was studied by simultaneous differential thermal and thermogravimetric analyses and the results are presented in Figure 4. According to the literature [7,8,9,12,13], one should first observe the loss of adsorbed water (1), then the loss of water in the channels (2), and, finally, the transformation of rhabdophane into monazite (3). Most of the literature places channel water loss in the temperature range between 110 and 245 °C, while irreversible exothermic transformation of rhabdophane into monazite is stated to occur at temperatures above 600 °C [10,13]. The following reactions are expected to take place:

CePO_4_∙nH_2_O (channels) mH_2_O (adsorbed) → CePO_4_∙nH_2_O + mH_2_O↑ (1)

CePO_4_∙nH_2_O → CePO_4_ + nH_2_O↑ (2)

CePO_4_ (rhabdophane) → CePO_4_ (monazite) (3)

According to Figure 4, the total mass loss between room temperature and 1000 °C was 10.36%. It is also evident that there is one broad endothermic peak in the range between room temperature and roughly 400 °C, as well as a small exothermic peak at ~600 °C. The DTG curve indicates that, in the interval from room temperature to 400 °C, two processes accompanied with mass loss occur. The first one is between room temperature and approximately 125 °C, accompanied with a mass loss of 3.42%, which is a consequence of the evaporation of adsorbed water. The next temperature range where significant mass loss occurs can roughly be placed between 125 and 400 °C, where 6.4% of mass is lost. This mass loss is the result of the conversion of hydrated into anhydrous rhabdophane due to the release of water molecules from the channels of rhabdophane’s structure. The sample loses an additional 0.5% of mass until 1000 °C. Matrasek et al. observed a mass loss at 900 °C in a similar system and have given an interesting analysis of possible causes for this phenomenon but have also given good arguments for rejecting each of them [10]. The mass fraction of water in rhabdophane (assuming the formula CePO_4_∙H_2_O) is 7.12%. With the mass correction considering the adsorbed water (3.42%), the expected loss of water located in the channels is 6.87%. This value roughly corresponds to the measured loss in the 125–400 °C range (6.4%) and matches almost exactly when the mass loss in the range between 400 and 1000 °C (0.5%) is included.

Upon varying the thermal treatment temperature, it is possible to confirm the assumptions on thermally induced events noticed via DTA/TGA analysis. Based on the DTA/TGA analysis results, temperatures of 300, 600, and 900 °C were chosen as temperatures the sample was thermally treated to and analyzed using XRD and FTIR analyses.

**Figure 5:**
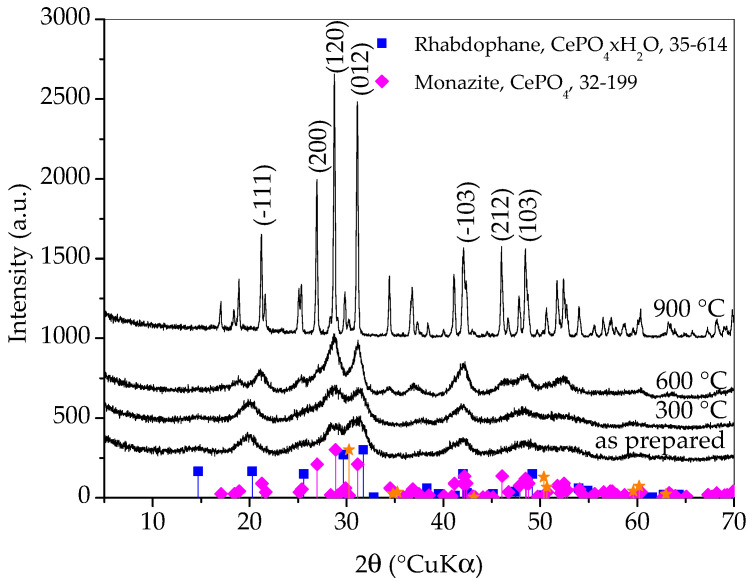
X-ray diffraction patterns of samples prepared by 15 min synthesis and heated to various temperatures. Only main monazite peaks are labeled. The yellow stars indicate the angles where the zirconia peaks (ICDD PDF No. 50-0189) would appear.

From Figure 5, it can be observed that the diffraction patterns of as-prepared sample and sample heated to 300 °C are quite similar and it is safe to say that the sample heated to 300 °C is still composed dominantly of rhabdophane. In the diffraction pattern of sample heated to 600 °C, the transformation of rhabdophane into monazite is clearly visible. The diffraction peaks are still very broad and the change does not seem dramatic at first glance, but a careful look at the pattern will make it clear that the transformation of rhabdophane into monazite is complete at 600 °C. Thermal treatment to 900 °C caused the growth of monazite crystallites, which is evident from the distinct narrowing of the diffraction maxima, suggesting an increase in monazite crystallite size. It is possible that a zirconia (011) peak (at 30.25 °2θ) emerged in the XRD pattern of the sample heated to this temperature. However, at the same angle, the (−211) peak of monazite should appear. Other zirconia peaks have lower intensities and also overlap with monazite peaks. From the obtained results, it can be concluded that the prepared rhabdophane is quite thermally stable but, despite the literature sources, the phase transformation of rhabdophane into monazite occurs at a temperature below 600 °C. Based on this analysis, a weak exothermic peak appearing on the DTA curve around 600 °C could be attributed to the transformation of rhabdophane to monazite.

**Figure 6:**
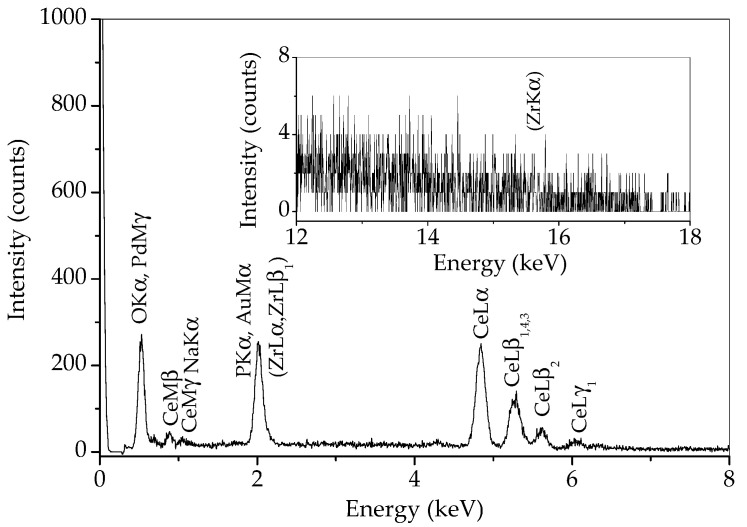
EDS spectrum of sample prepared by 15 min synthesis.

In order to determine whether there is any zirconia in the sample prepared by 15 min synthesis, an EDS analysis of a wide area of this sample was performed and the result is given in Figure 6. Some of the characteristic Zr lines (L lines) are overlapped with characteristic lines for phosphorus and gold from conductive coating. However, if zirconium is present, the ZrKα line at 15.77 should be visible. As can be observed in the inset in Figure 6, no line appears at this energy. Therefore, the peak at 30.25 °2θ most likely belongs to monazite, and zirconium is either absent in this sample or present in a minimal amount.

**Figure 7:**
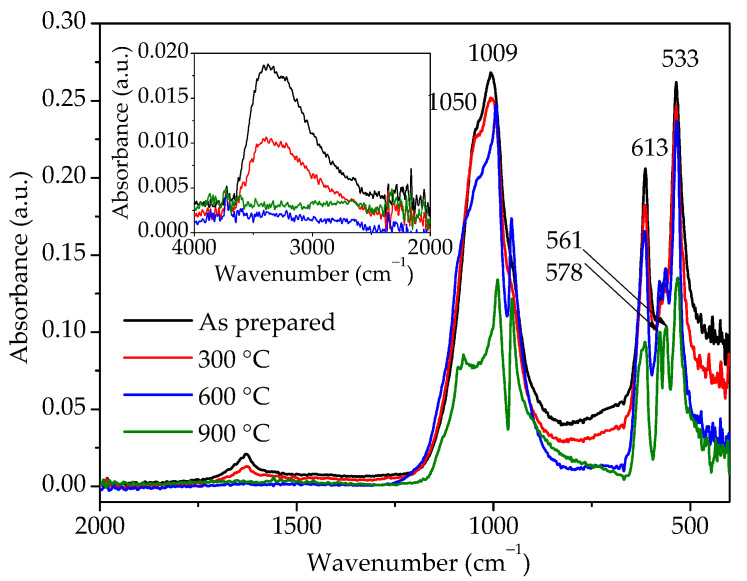
FTIR spectra of samples prepared by 15 min synthesis and heated to various temperatures.

FTIR analyses were conducted for the same samples and the spectra are shown in Figure 7. The broad band between 3750 and 2250 cm^−1^ and a small band at 1630 cm^−1^ could be observed for the as-prepared sample. The first band is due to –OH stretching, while the second one is attributed to –OH bending mode [12,13]. The diminished intensity of these two bands in the sample heated to 300 °C clearly indicates that the amount of water in this sample is reduced, while, after heating to 600 °C, there is no more water in the sample. IR spectra of the as-prepared sample and sample heated to 300 °C show the presence of bands at 533 and 613 cm^−1^ and a band at 1009 cm^−1^ with a shoulder at 1050 cm^−1^ characteristic for the hydrous hexagonal orthophosphates [10,20], which correspond to O–P–O bending, O=P–O bending, and P–O stretching vibration modes, respectively [8,11,13]. On the other hand, samples heated to 600 and 900 °C show band splitting in the wavenumber ranges of 950–1100 and 500–600 cm^−1^, typical for monoclinic phosphates [2,9,10]. Therefore, judging by the FTIR spectra, i.e., the splitting of bands in the region characteristic for PO_4_^3-^ groups’ vibrations and bands related to the presence of water, hexagonal rhabdophane is present in the as-prepared sample and sample heated to 300 °C, while, in the samples heated at 600 and 900 °C, the presence of monoclinic monazite is confirmed.

**Figure 8:**
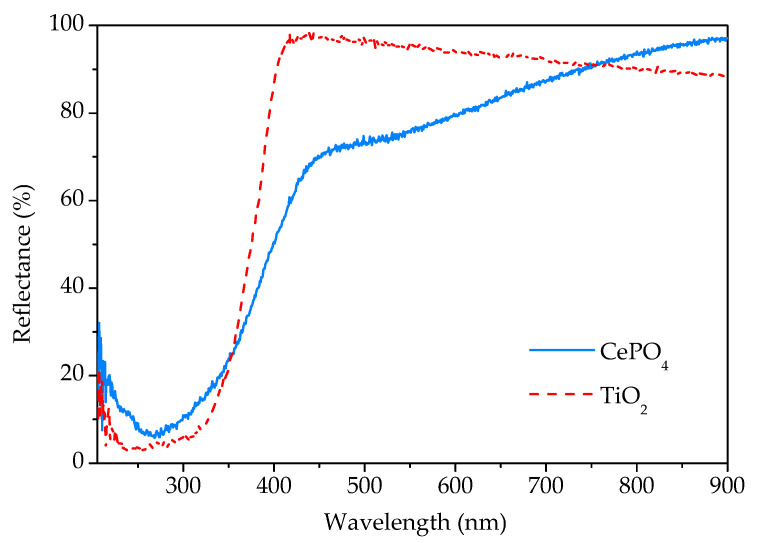
UV-Vis diffuse reflectance spectra of phosphate sample prepared by 15 min synthesis and commercial titania.

The UV-Vis DRS spectrum of the sample prepared by 15 min synthesis in comparison with the commercial TiO_2_ spectrum is given in Figure 8. As can be seen, the sample reflects a large part of the radiation of the visible spectrum. In the vicinity of the transition from the visible to the UV area, there is a sudden decrease in reflectance due to the absorption of UV radiation. According to the literature [3,21], rhabdophane is a direct bandgap material. From the Tauc’s plot, the energy of the bandgap for the direct transition was determined to be 3.1 eV, which is close to the value of 2.7 eV obtained by Lima et al. [3] in their study of rhabdophane-type CePO_4_ nanoparticles.

**Figure 9:**
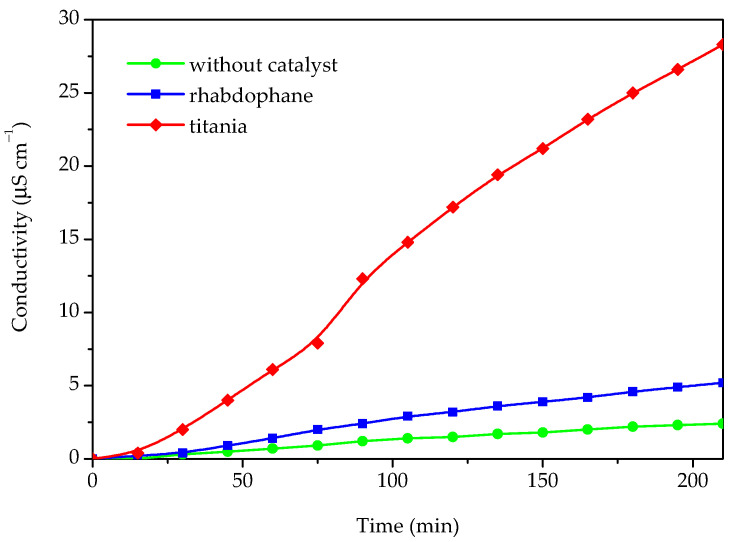
The conductivity of Castor oil degradation products dissolved in water after photocatalytic degradation under UV light without any catalyst and in presence of phosphate sample and commercial titania.

In order to evaluate the possibility of utilizing the prepared material in photoprotective formulations, the photocatalytic activity for the Castor oil degradation process was investigated (Figure 9).

The photocatalytic activity was determined by measuring the conductivity of the water containing the volatile degradation products of the Castor oil degradation process. For the purpose of comparison, the same experiment was accomplished in the presence of commercial titania and without any catalyst. The results of the photocatalytic degradation experiments, i.e., conductivity as a function of time, are presented in Figure 9. As can be seen from Figure 9, some catalytic activity of the investigated sample could be observed because the conductivity in the presence of the investigated sample is roughly twice as high as without any catalyst. However, in comparison with titania, which has a significantly higher activity, the photocatalytic activity of the prepared material could be rated as very weak. Based on the conducted photocatalytic experiment, it can be concluded that the investigated material could be suitable for use as a UV filter in sunscreens since it behaves as a nonoxidizing agent and does not impair sunscreen photostability.

**Figure 10:**
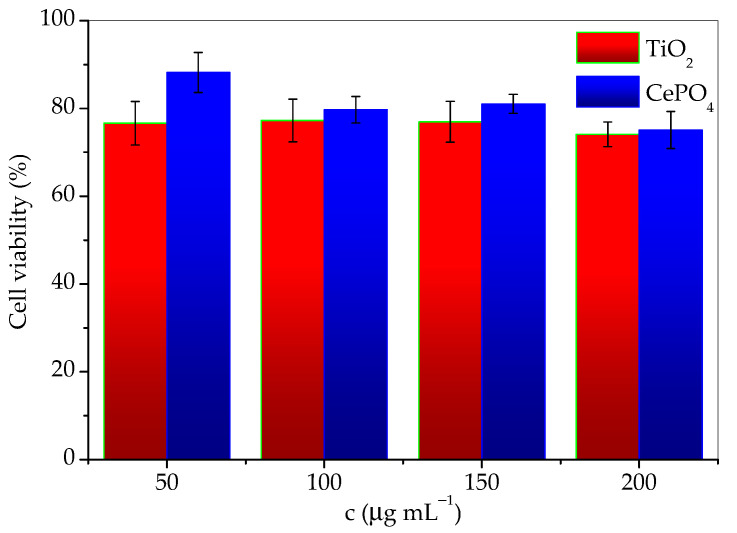
Cell viability as a function of TiO_2_ and CePO_4_ concentration.

The results of cytotoxicity investigation of the prepared sample and commercial titania, expressed as the percentage of surviving cells depending on the concentration are given in Table 1. The data are also given in Figure 10 for easier comparison. According to the guidelines for the determination of in vitro cytotoxicity of medicinal products, a material is considered nontoxic if cell survival is ≥70% after treatment. From the tabular data of the obtained results, it can be seen that the cells tolerated the treatment of both agents well, and the survival percentage was satisfied by both materials. It can be concluded that both TiO_2_ and CePO_4_ are noncytotoxic and suitable for use in skin products. However, although the differences in values are small, it should be emphasized that cell survival was greater in the presence of the investigated phosphate sample. This difference was especially notable for the lowest concentrations. It should be noted that cell viability decreases slightly faster with concentration in the presence of CePO_4_ than TiO_2_. Therefore, conducting the cytotoxicity research in a wider concentrations range would be advisable.

## 3. Materials and Methods

Cerium phosphate was prepared by mechanochemical synthesis in a Pulverisette 6 planetary mill (Fritsch, Idar-Oberstein, Germany) using an 80 mL zirconium oxide (ZrO_2_) vessel and 10 mm diameter ZrO_2_ spheres (total mass of spheres = 47.38 g). Cerium (III) chloride heptahydrate, CeCl_3_∙7H_2_O (Acros Organics, Geel, Belgium, 99%), and sodium phosphate dodecahydrate, Na_3_PO_4_∙12H_2_O (Kemika, Zagreb, Croatia, 99%), were used as reactants. In order to prevent the growth of cerium phosphate particles, sodium chloride, NaCl (Lach-ner, Zagreb, Croatia, 99%), was added to the reaction mixture as a diluent. The mass ratio of balls and powder was 6:1; it follows that the mass of the powder was 7.97 g. The ratio of NaCl and precursors was also 6:1, so the mass of NaCl was 6.8336 g and the total mass of precursors was 1.1393 g (0.5642 g CeCl_3_∙7H_2_O and 0.5751 g Na_3_PO_4_∙12H_2_O). Syntheses were carried out for 60, 30, and 15 min at 600 rpm. After synthesis, to wash away NaCl, the resulting powder was mixed with 200 mL of distilled water, sonicated in an ultrasonic bath for five minutes, and then centrifuged at 3500 rpm for five minutes, followed by decantation. The procedure was repeated five times. Finally, the resulting powder was dried in a laboratory oven at 105 °C for 24 h. Part of the material remained on the vessel walls and on the balls and part was lost in the washing procedure, which resulted in a yield of only 50–60%.

X-ray diffraction was performed on a Shimadzu (Tokyo, Japan) XRD 6000 diffractometer with CuKα radiation. Data were collected in the range from 5 to 70 °2θ with a step of 0.02° and a dwell time of 0.6 s. Crystallite size was calculated via Scherrer equation as described in [22]. Micrographs were obtained using Cs corrected high-resolution scanning transmission electron microscope (HR-TEM) Jeol (Tokyo, Japan) ARM 200 CF with accelerating voltage of 80 kV coupled with a Jeol Centurio 100 energy-dispersive X-ray spectrometry (EDS) detector providing elemental mapping. The EDS spectrum was obtained utilizing energy-dispersive X-ray spectrometer (EDS) Oxford INCA X-sight coupled with Tescan (Brno, Czech Republic) Vega3 EasyProbe scanning electron microscope. For the calculation of ceria average particle size, Image J software (https://imagej.net/, accessed on 12 December 2023) package was used [23]. Thermal analyses were performed on NETZSCH (Selb, Germany) STA 409 device with α-Al_2_O_3_ as a reference substance. About 50 mg of the sample was placed in a platinum container and heated at a rate of 10 °C min^−1^ with an air flow of 30 cm^3^ min^−1^. Fourier transform infrared spectroscopy analysis was accomplished on an ATR Bruker (Billerica, MA, USA) Vertex 70 device in the spectrum range 4000–400 cm^−1^, with a resolution of 2 cm^−1^. UV-Vis analysis was performed in the range of 200–900 nm on Ocean Insight (Orlando, FL, USA) QE Pro High-Performance spectrometer equipped with an integrating sphere for reflection. The bandgap energy was determined from the obtained spectrum using Tauc’s plot as described in [22].

**Figure 11:**
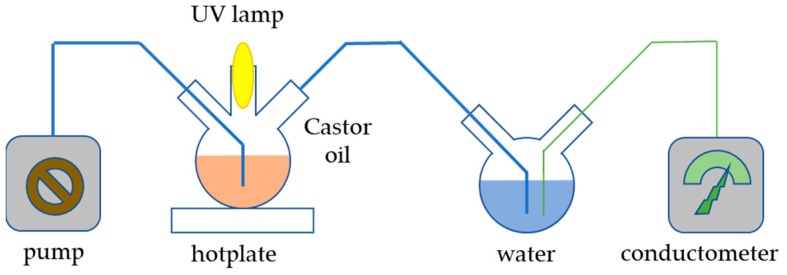
Scheme of the apparatus for the photocatalytic experiment.

In order to determine the potential photocatalytic activity of the prepared sample, which might compromise its applicability in sunscreen formulations, the Castor oil oxidation process under UV light was conducted. The setup of the experiment is depicted in Figure 11; 200 mg of catalyst was added to 20 mL of Castor oil and sonicated in an ultrasonic bath for 3 min. The prepared suspension was added to a flask placed on a hotplate. The content of the flask was continuously stirred and irradiated with 253 nm irradiation produced by a UV lamp placed in a quartz cuvette. The entire reaction system was isolated from an external source of radiation and blown with air using an electric pump so that the air stream could carry volatile products of Castor oil oxidation into a vessel filled with 150 mL of demineralized water. The dissolution of gaseous compounds in demineralized water caused a rise in conductivity, which was measured by a conductometer. Aside from the prepared material, the experiment was also conducted with commercial TiO_2_ nanoparticles for comparison and without any catalyst.

Cytotoxicity was studied using the human keratinocyte cell line (HaCaT), provided by Cell Line Services, Eppelheim, Germany. HaCaT were supplemented with 10% bovine serum and a mixture of penicillin, streptomycin, and amphotericin B. The cells were seeded in 96 wells of a plate, with each well containing 104 cells, and left for 48 h to grow. Then, the medium was removed and cells were washed and exposed to the prepared material for a period of 24 h. After this time, in vitro cytotoxicity was determined using the MTT test, a nonradioactive colorimetric test based on the reduction of the yellow tetrazolium salt (3-(4.5-dimethylthiazol-2-yl)-2.5-diphenyltetrazolium bromide (MTT) to purple formazan crystals by metabolically active cells. Living cells contain NAD(P)H-dependent oxidoreductase enzymes, which reduce MTT to formazan. The obtained crystals are dissolved, and the resulting colored solution is quantified by measuring the absorbance in the wavelength range of 500–600 nm using a multi-well spectrophotometer [24]. The cytotoxicity of commercial TiO_2_ nanoparticles was determined for the purpose of comparison.

## 4. Conclusions

Nanoparticles of rhabdophane (CePO_4_∙H_2_O) were prepared by mechanochemical synthesis from cerium (III) chloride heptahydrate and sodium phosphate dodecahydrate with sodium chloride as diluent. The duration of 15 min proved to be the best in order to ensure the completion of the process and avoid contamination of the sample with vessel material. The size of the prepared rhabdophane nanoparticles was around 5 nm. Thermal analysis combined with XRD and FTIR analyses showed the elimination of adsorbed and channel water, and, finally, the transformation of rhabdophane to monazite at around 600 °C. DRS analysis showed that the prepared material absorbs UV rays, while the absorption in the visible area is minimal. It was established that the photocatalytic activity of the prepared nanoparticles in the process of Castor oil oxidation was weak. The prepared rhabdophane nanoparticles were proven to be noncytotoxic for the human keratinocyte cells. Based on all of the above, the prepared rhabdophane can be evaluated as a promising UV filter in photoprotective formulations.

## Figures and Tables

**Table 1:** The comparison of prepared phosphate and commercial titania cytotoxicity.

Concentration (μg mL^−1^)	Cell Survival with TiO_2_ (%)	SD	Cell Survival with CePO_4_ (%)	SD
50	77	5	88	5
100	77	5	80	3
150	77	5	81	2
200	74	3	75	4

## Data Availability

The original contributions presented in the study are included in the article; further inquiries can be directed to the corresponding author.
